# Influence of plastic aggregate geometry on strength properties of cement-stabilized macadam

**DOI:** 10.1038/s41598-023-41453-2

**Published:** 2023-09-14

**Authors:** Mingxiang Chi, Zhenzong Shen, Shibin Chen, Yunshi Yao, Jianjun Shen, Chao Li, Qingyao Yan

**Affiliations:** 1 Key Laboratory of Road Construction Technology and Equipment of MOE, Chang’an University, Xi’an, 710064 China; 2 Shandong Hi-Speed Engineering Construction Group Co., Ltd., Jinan, 250014 Shandong China

**Keywords:** Civil engineering, Mechanical engineering

## Abstract

In order to improve the toughness and flexibility of cement-stabilized macadam and inhibit crack formation and propagation, the influence of plastic aggregate geometry on the strength properties of cement-stabilized macadam is studied. The surface roughness of plastic aggregate was designed into Ra 12.5 and Ra 25. When the content of plastic coarse aggregate was gradually increased from 4 to 8%, 12%, 16%, 20%, and 24%, the compressive strength, splitting strength, and the tensile-compression ratio of the cement-stabilized macadam made of aggregate with rough surface texture were found higher than those of aggregate with smooth surface texture. The rough cylindrical, spherical, spindle, and dumbbell-shaped plastic coarse aggregates were designed and prepared for testing. The test results showed the maximum compressive strength, splitting strength, and tensile-compression ratio of cement-stabilized macadam with the dumbbell-shaped plastic coarse aggregate. The tensile-compression ratio increased by 15.2% compared to natural aggregate cement-stabilized macadam. The results show that under the same conditions, the plastic aggregate with different geometry can improve the compressive and splitting strengths of cement-stabilized macadam and significantly improve the toughness and flexibility, with dumbbell-shaped plastic coarse aggregate having the greatest improvement, especially compared with natural aggregate cement-stabilized crushed stone. This research provides insights for improving pavement engineering quality and new ideas for utilizing waste plastics.

## Introduction

The cement-stabilized macadam semi-rigid base has high stiffness, water stability, and frost resistance. Its high bearing capacity is less affected by water and freeze–thaw cycles, reducing the bending and tensile stress of asphalt pavement^1–3^. Hence, it is widely used in hierarchical roads. However, the existing semi-rigid base layer has poor toughness and is prone to cracking. With the extension of the road service life, the cracks often extend to the surface layer, eventually forming large macroscopic cracks through the road, affecting the service life of the road and endangering traffic safety^4–6^. Therefore, it is of great significance to improve the pavement performance of the cement-stabilized macadam base material and reduce the early fatigue damage of the pavement structure. In order to improve the toughness of cement-stabilized macadam and inhibit the formation of cracks, many methods have been applied in engineering. Usually, cement-based materials are mostly reinforced by adding steel or polypropylene fibers^7–10^. However, steel and polypropylene fibers are not easily mixed with cementitious materials evenly during the mixing process. Also, the amount used is not large, which only accounts for 1.5–3% of the total mixture volume, and the improvement effect on toughness is less than 10%.^11,12^. Therefore, the exploration of flexible alternative aggregates has become the research focus.

Plastic has become one of the four basic materials in the world today because of its light weight, convenient processing, and economical and practical characteristics^13,14^. A large amount of plastic waste is produced annually worldwide. According to statistics, the total amount of waste plastics recycled in China in 2020 reached 20 million tons, which caused serious environmental pollution and endangered human health. Currently, waste plastics are mostly treated by dumping in landfills or centralized incineration. However, such methods still cause secondary pollution. Thus, plastic waste recycling has become a dire need. Recycling waste plastics as new aggregates in road materials is one of the best solutions to address this issue^15–18^.

Brahim^19^ pulverized polypropylene plastic into fine particles and mixed it into concrete as a sand replacement. The results of mechanical test showed that the compressive strength of self-comp acting mortar containing up to 50% of plastic waste was acceptable for lightweight mortars. Yazoghl^20^ pulverized waste PET into fine aggregate particles of three particle sizes and replaced fine sand with different volume ratios. The resulting mechanical properties were not significantly affected when the replacement rate was less than 50%; however, a significant change was detected for the higher replacement rate. Nabajyoti and Saikia^21,22^ studied the effect of coarse aggregate replacement with PET plastic on concrete compressive and tensile strengths. The incorporation of plastic improved the concrete toughness, while the compressive and splitting tensile strength degradations were also proportional to the replacement rate. Choi^23^ used fibers derived from waste plastic bottles and mixed them into concrete. It was demonstrated that the concrete specimens mixed with plastic had better ductility than those without plastic, the workability of concrete with 75% waste PET aggregate improves about 123% compared to that of the normal concrete and the crack expansion was effectively inhibited.

The above studies show that even though incorporating plastic aggregates into cement-based materials reduces the strength index of cement-based materials, it increases their toughness and ductility. If the toughness and ductility are effectively improved while meeting the strength requirements, it can inhibit or delay the cracking of cement-stabilized macadam. Also, it can better handle waste plastics and protect the environment. However, there are limited studies on waste plastics incorporation in cement-stabilized macadam. The influence of geometric characteristics of plastic aggregate on the performance of cement stabilized macadam is often neglected.

In addition to the strength of the aggregate itself, the mechanical strength of the mixture also includes the bond strength between the aggregate and gel, and the internal friction between the coarse aggregates. The geometry of the coarse aggregate is an important condition to ensure the internal friction between the aggregates, but also as an important indicator to determine whether the base material can constitute a good skeleton, whether the formation of embedded between the aggregates.

Unlike natural aggregates, the geometric characteristics of plastic aggregates could be designed. Therefore, in this paper, the strength properties of cement-stabilized macadam incorporating waste plastic aggregates have been evaluated. The impact of geometry of plastic aggregates on the strength index and toughness has been studied, including roughness of surface texture and shape of the plastic aggregates. The study suggests new materials for pavement construction and new methods for waste plastic disposal.

## Materials and experimental methods

### Raw materials and mix proportions

In this study, the experimental work was designed for semi-rigid base cement-stabilized macadam. The effect of different surface roughness and geometry of plastic coarse aggregate on strength properties of cement-stabilized macadam was investigated by adding plastic particles as coarse aggregate to replace natural coarse stone by equal volume. The cement used in the test is labeled P.O 32.5. Rod-shaped polyvinyl chloride (PVC) plastic was used as raw material in the test, and its basic mechanical properties are shown in Table 1. The specific specifications of the PVC rod purchased are φ10 × 1000 mm and φ15 × 1000 mm.

**Table 1:** Basic mechanical properties of PVC plastics.

Size deviation/mm	Density/g/cm^3^	Tensile strength/MPa	Elongation at break/%	Corrosion/g/m^2^
0.2	1.54	27	4.1	− 4.6

**Figure 1:**
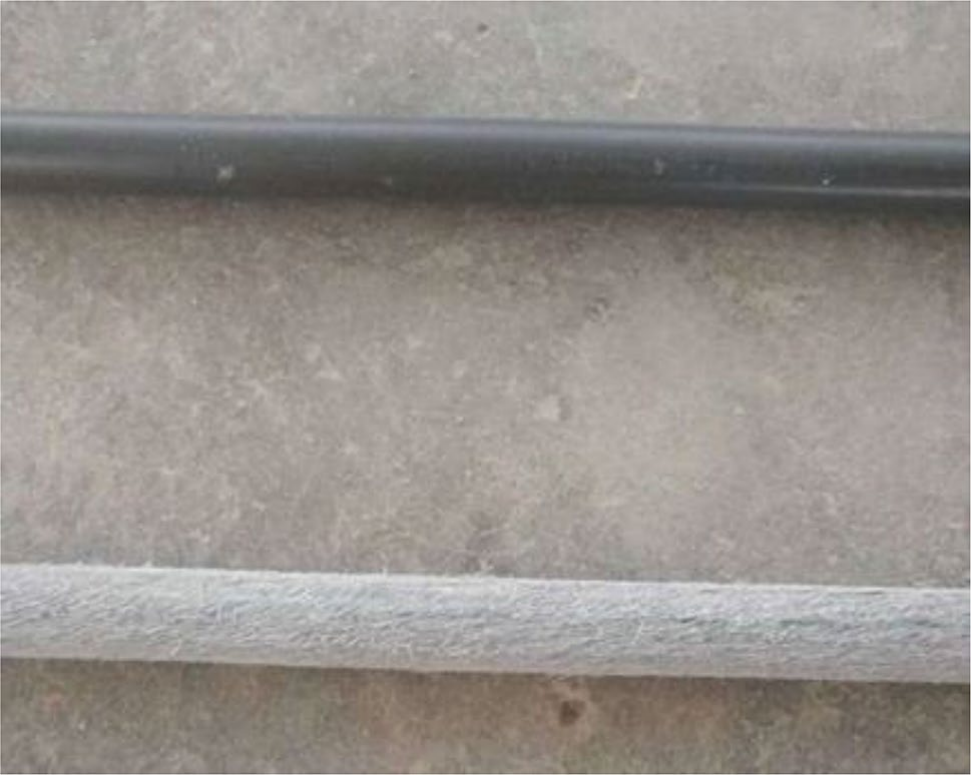
Plastic rod with different roughness.

The preparation of plastic coarse aggregates for experiments was carried out according to different experimental conditions. The 10mm diameter plastic rod was roughened with a file so that the surface roughness between Ra12.5 and Ra25 could be achieved. The cutting equipment was used to cut into sections, and the length of each section was 15–20 mm. It can be seen from Fig. 1 that the surface of the plastic rod after rough treatment is not smooth and has fibrous burrs.

**Figure 2:**
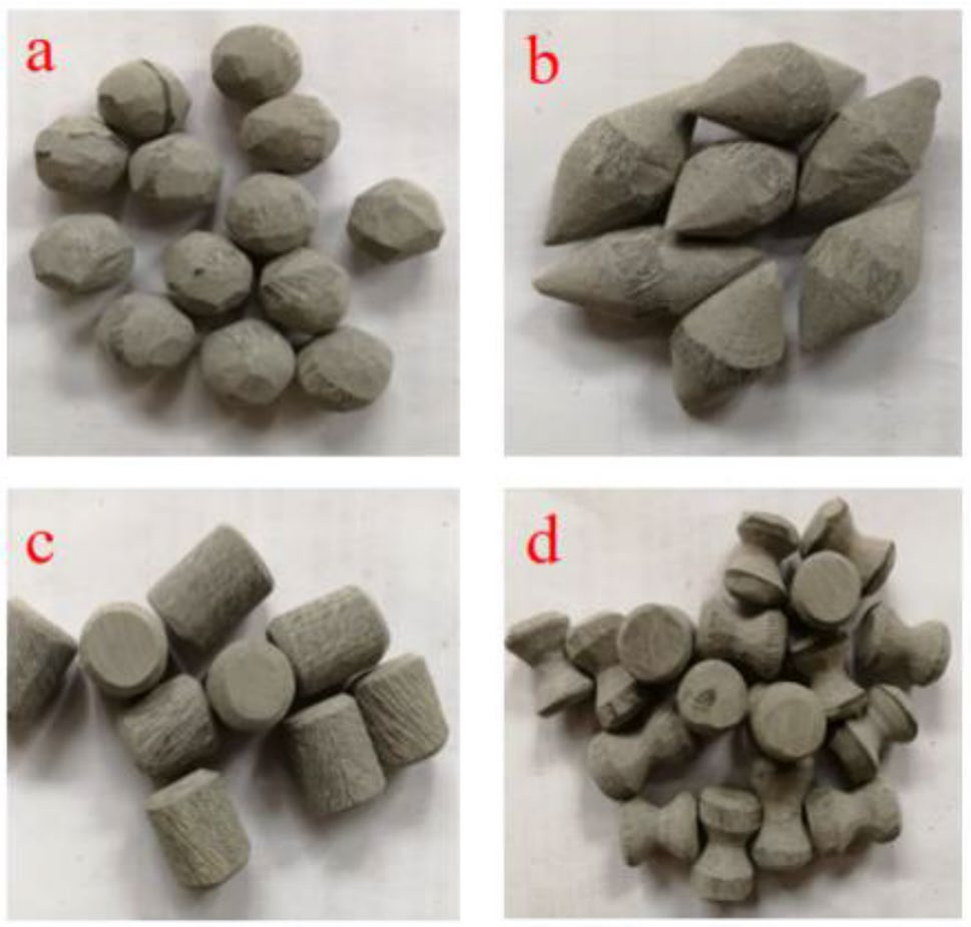
Plastic coarse aggregates of different shapes; (**a**) spherical plastic coarse aggregate; (**b**) spindle-shaped plastic coarse aggregate; (**c**) cylindrical plastic coarse aggregate; (**d**) dumbbell-shaped plastic coarse aggregate.

The plastic rod with a diameter of 15 mm was first cut into segments. Then, the plastic coarse aggregates of different shapes were prepared by grinding. The prepared aggregate is shown in the Fig. 2. The plastic was ground into spherical, spindle, cylindrical, and dumbbell shapes to ensure a distinct and characteristic distinction between aggregates of each shape. These four shapes reflect the macroscopic overall changing characteristics and state of the plastic coarse aggregate respectively. Dumbbell-shaped plastic coarse aggregate is processed on the basis of cylindrical plastic coarse aggregate and processed into a concave shape in the middle; spindle-shaped plastic coarse aggregate is processed on the basis of cylindrical plastic coarse aggregate and ground into a cone shape at both ends; spherical plastic coarse aggregate has the largest sphericity among the four types of plastic coarse aggregate and has the most angles; cylindrical plastic coarse aggregate is simply processed for surface texture.

The aggregate for control specimens used in the test was limestone. Limestone aggregate size was divided into three grades: 0–5 mm, 5–10 mm, and 10–20 mm. According to the “Test Methods of Aggregate for Highway Engineering JTG E42-2005”, the suspension dense grade was chosen to form the mixture. The mass ratio of the three kinds of aggregates was (10–20 mm): (5–10 mm): (0–5 mm) = 28.56: 37.13: 34.31. The specific mix proportion is shown in Table 2.

**Table 2:** Mix proportions of cement-stabilized macadam.

Aggregate specifications (mm)	Mass percentage	The mass percentage (%) passing through the following square mesh sieve (mm)						
		31.5	19.0	9.5	4.75	2.36	0.6	0.075
10–20	28.56%	28.56	25.42	0.07	0.07	0.07	0.05	0.04
5–10	37.13%	37.13	37.13	36.44	1.82	0.10	0.08	0.05
0–5	34.31%	34.31	34.31	34.31	34.19	25.53	14.17	0.55
Synthetic proportion		100	96.86	70.81	36.07	25.70	14.3	0.64
Upper limit		100	100	90	49	32	20	5
Lower limit		100	90	60	29	15	6	0

### Specimen preparation

**Figure 3:**
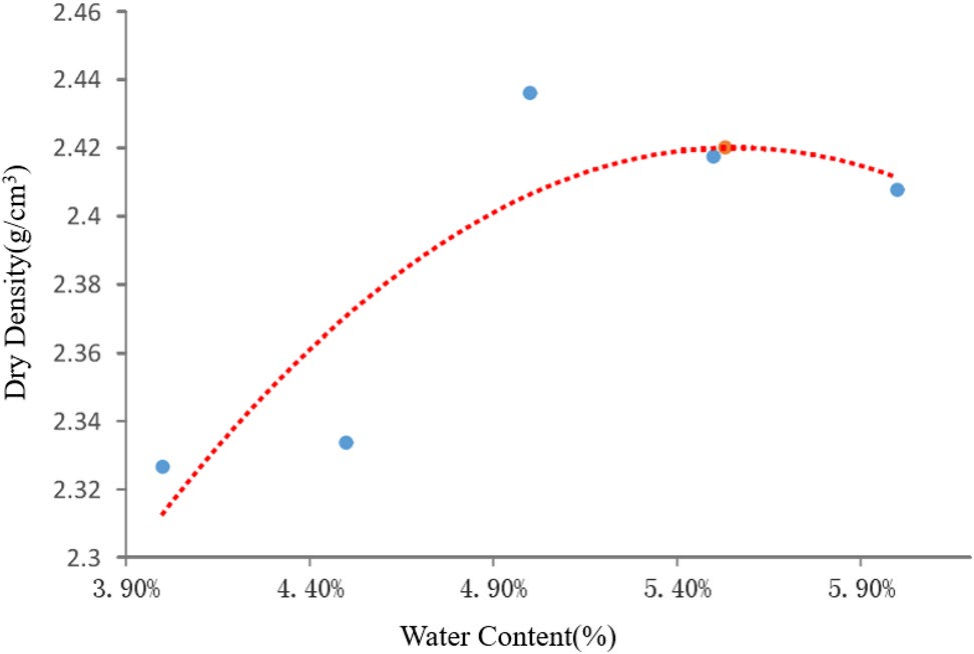
The results of compaction test.

In order to determine the water content of the mixture, the optimum water content and maximum dry density of the mixture were determined by compaction test. Ordinary tap water was used for mixing. The standard proctor was used to carry out the compaction test, with an average unit compaction work of 2.687 J. Figure 3 shows the results of compaction test and the fitted curve. From the compaction test results, the optimum water content was found as 5.53%, and the correspondingongg maximum dry density was 2.42 g/cm^3^. The cement content was 4.6%.

The plastic content was set to replace 10–20 mm coarse aggregate with an equal volume, and the replacement rate was 4%, 8%, 12%, 16%, 20%, and 24%. In the test of the different shapes of plastic coarse aggregate, the 10–20 mm coarse aggregate was replaced by plastic aggregate at the equivalent volume, and the replacement rate was 16%. According to the “Test Methods of Materials Stabilized with Inorganic Binders for Highway Engineering JTG E51-2009”, the φ100 × 100 cylindrical specimens were prepared in both experiments, which were cured for 28 days under standard conditions, and their unconfined compressive and splitting strengths were measured. Nine specimens were used for each test to ensure the statistical significance of the test results.

## Results

### Influence of surface roughness of plastic coarse aggregate on strength properties

Waste plastics have a small elastic modulus, smooth surface, and weak interface bonding. In order to enhance the strength properties of plastic aggregate cement-stabilized macadam, the surface of plastic aggregate was treated to Ra12.5–Ra25 to prepare cement-stabilized macadam. It is clear from Table 3 that, compared with natural stone, the average splitting strength and compressive strength of cement-stabilized macadam decrease after adding plastic aggregates and tend to decrease with the increase in plastic content. When the amount of plastic coarse aggregate is gradually increased from 0 to 4%, 8%, 12%, 16%, 20%, and 24%, under the same amount of plastic added, the compressive strength of the specimens incorporated with rough plastic coarse aggregates increased by 2.2%, 3.2%, 5%, 2.4%, 1.1%, and 0.5%, respectively, compared to the specimens with smooth surface plastics. The corresponding splitting strength and tension–compression ratio increased by 3.9–5.7% and 1.6–5.0%, respectively.

**Table 3:** Strength properties of cement-stabilized macadam adding different roughness plastic aggregates.

Plastic content/%	Surface properties	Average splitting strength/MPa	Average compressive strength/MPa	Tension–compression ratio (%)
0	None	0.65	10.53	6.19
4	Smooth	0.62	9.70	6.38
	Rough	0.64	9.91	6.48
8	Smooth	0.59	9.43	6.22
	Rough	0.63	9.73	6.47
12	Smooth	0.53	8.57	6.20
	Rough	0.58	9.00	6.50
16	Smooth	0.52	8.21	6.31
	Rough	0.57	8.41	6.75
20	Smooth	0.48	7.86	6.15
	Rough	0.51	7.95	6.36
24	Smooth	0.46	7.65	5.9
	Rough	0.48	7.69	6.25

The test results show that the cement-stabilized macadam mixed with surface-treated plastic aggregates indicates better strength properties (compressive and splitting strength) and toughness (tensile-compression ratio). The main reason could be assumed that the interfacial transition zone (ITZ) between the mortar and the plastic coarse aggregate is a weak link in the semi-rigid base, and the strength of the contact interface is generally lower than the strength of the aggregate and the mortar itself. Compared with the plastic coarse aggregate with a smooth surface, many capillary filament-like plastic fibers are produced after the plastic surface is roughened. These filamentous plastic fibers on surfaces can combine with the cement mortar, so the mortar is firmly attached to the plastic aggregate surface. When the specimen is damaged by loading, the strength of its interface is higher than before, and the propagation speed of the micro-crack becomes slower, making it more difficult to be damaged.

### Influence of shape of plastic coarse aggregate on strength properties of cement-stabilized macadam

#### Compressive and splitting tensile strength

**Figure 4:**
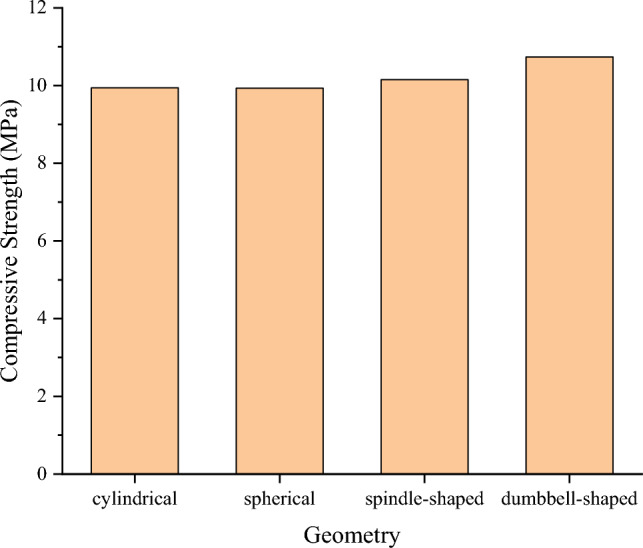
Influence of different shapes of plastic coarse aggregate on the compressive strength.

The particle size of the plastic coarse aggregate used in the test is 15mm, and the surface is rough-treated. Figure 4 shows the influence of different plastic coarse aggregate shapes on the specimen’s compressive strength. It can be seen from Fig. 4 that the compressive strengths of the specimens mixed with cylindrical, spherical, spindle-shaped, and dumbbell-shaped plastic coarse aggregates are 9.492 MPa, 9.933 MPa, 10.15 MPa, and 10.738 MPa, respectively. The compressive strength of specimens with dumbbell-shaped plastic coarse aggregates is 5.81% higher than those containing spindle-shaped plastic aggregates. Also, the specimens incorporated with the spindle-shaped plastic coarse aggregate specimen show 2.21% higher compressive strength than those containing spherical-shaped plastic aggregates. The compressive strength of the spherical plastic coarse aggregate specimens is 4.44% higher than that of the cylindrical shape. The compressive strength of the specimen with dumbbell-shaped plastic coarse aggregate is 13.17% higher than that of the specimen mixed with cylindrical plastic coarse aggregates.

**Figure 5:**
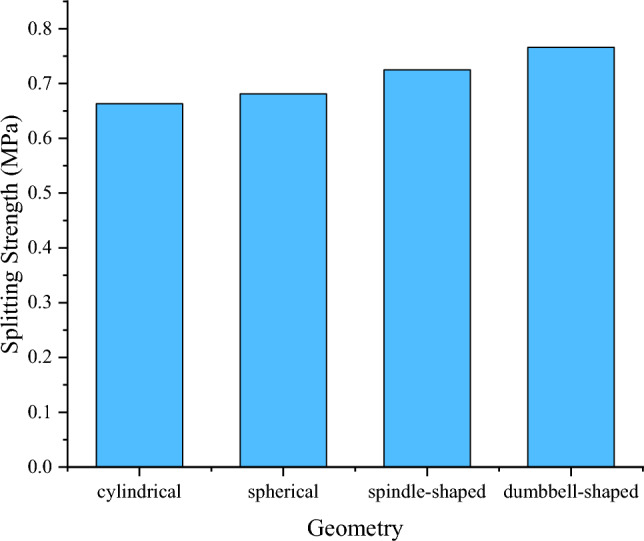
Effect of different shapes of plastic coarse aggregates on the splitting strength.

Figure 5 shows the effect of different shapes of plastic coarse aggregates on the splitting strength of the specimens. The splitting strength of the specimens mixed with cylindrical, spherical, spindle, and dumbbell-shaped plastic coarse aggregates were 0.663 MPa, 0.681 MPa, 0.725 MPa, and 0.766 MPa, respectively. The splitting strength of specimens incorporated with dumbbell-shaped plastic coarse aggregate is 5.65% higher than those containing spindle-shaped specimens. Also, the splitting strength of spindle-shaped plastic coarse aggregate specimens is 6.46% higher than those with spherical plastic aggregates. Further, the splitting strength of specimens prepared with spherical plastic coarse aggregate is 2.71% higher than those containing the cylindrical ones. The splitting strength of the specimen with dumbbell-shaped plastic coarse aggregate was increased by 15.53% compared with that of the specimen mixed with the cylindrical plastic coarse aggregates.

**Figure 6:**
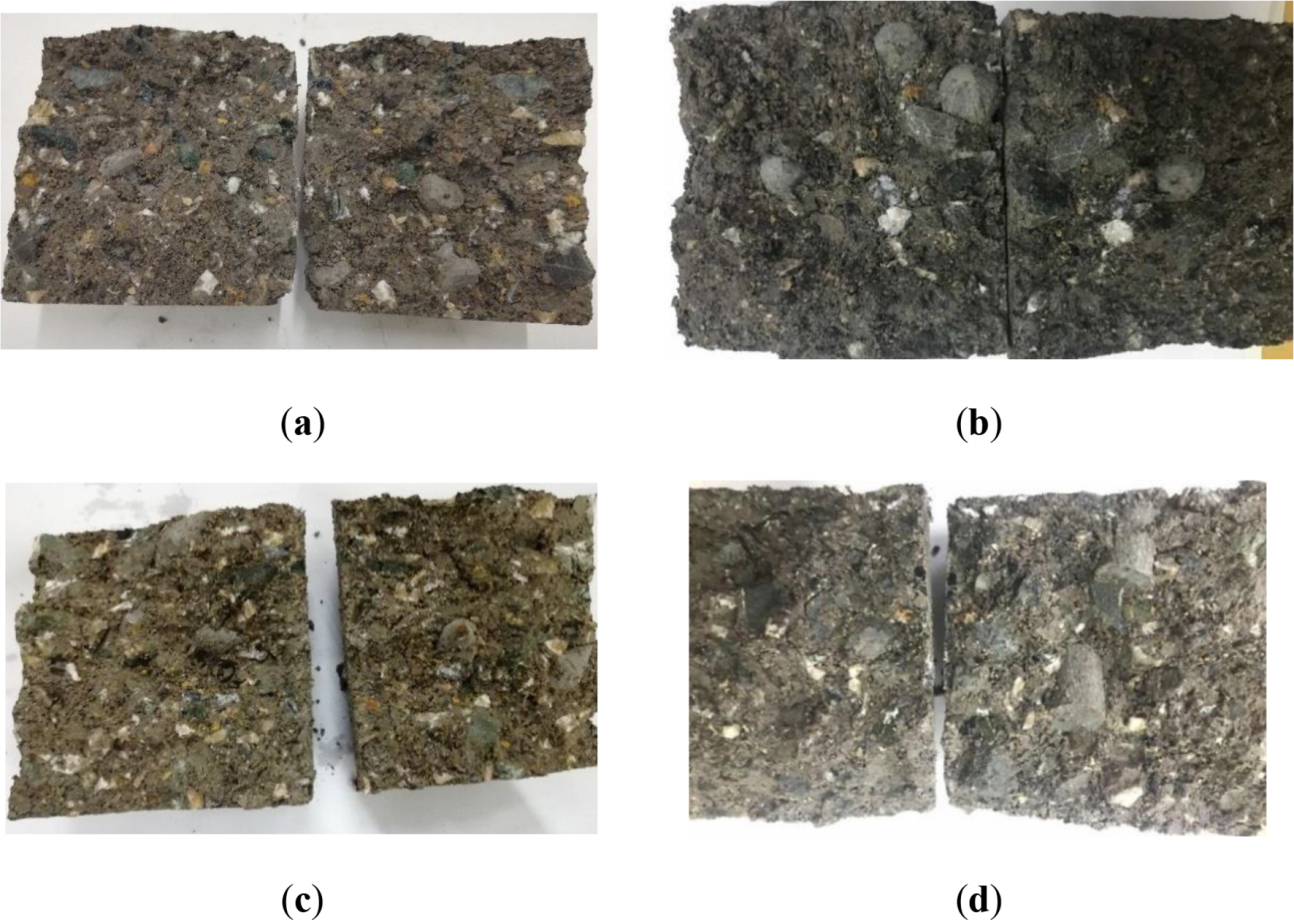
Morphology of plastic coarse aggregates with different shapes in the specimen: (**a**) dumbbell-shaped; (**b**) spherical; (**c**) spindle-shaped; (**d**) cylindrical.

Figure 6 shows the morphology of different shapes of plastic coarse aggregate in the specimen. It can be seen that the plastic aggregate is evenly distributed in the cement-stabilized macadam. The dumbbell-shaped plastic coarse aggregate is concave in the middle. Its special geometric structure can better adhere to cement mortar or other aggregates than the other three types of plastic aggregates. Thus, higher strength of the resulting composites is attained. The intensity is naturally higher. The spindle-shaped plastic coarse aggregate is conical at both ends, which is easier to “insert” into the mixture than the spherical and cylindrical plastic coarse aggregate. Hence, the performance of these two plastic shapes is better. Compared with dumbbell-shaped and spindle-shaped plastic coarse aggregates, spherical plastic coarse aggregates have smaller sphericity, leading to smaller contact interfaces between aggregates. Many contact surfaces may be line-to-line contact or even point-to-line contact. During the strength test, these weak areas are easily damaged, causing the spherical plastic coarse aggregate to be peeled off, and the overall strength of the test piece will be greatly reduced.

#### Tensile-compression ratio

Toughness is the ability of a material to absorb deformation forces when it deforms. Tensile-compression ratio is the ratio of splitting tensile strength to unconfined compressive strength. The magnitude of the tensile-compression ratio reflects the magnitude of the tensile strain energy per unit volume of the specimen under the compressive strain energy per unit volume. The tensile-compressive ratio indicates the energy absorbed per unit volume of the specimen during deformation, thus indirectly reflecting the toughness.

Table 4 shows the tensile-compression ratio of specimens mixed with different shapes of plastic coarse aggregates. It can be seen from Table 4 that the shape of the plastic coarse aggregate has a certain influence on the tension–compression ratio of the cement-stabilized macadam. The tensile-compression ratio of the specimens mixed with spindle-shaped plastic coarse aggregate is the largest, which is slightly higher than that of the specimens mixed with dumbbell-shaped plastic coarse aggregate and increases by 15.2% compared with the cement-stabilized macadam without plastic aggregate (i.e., control specimen). The unique shape of the two kinds of aggregate increases the tension–compression ratio of the cement-stabilized macadam and improves its toughness.

**Table 4:** Tensile-compression ratios of cement-stabilized macadam mixed with different shapes of plastic coarse aggregates.

Geometry	Average splitting strength/MPa	Average compressive strength/MPa	Tension–compression ratio (%)
None	0.65	10.53	6.19
Cylindrical	0.663	9.492	6.98
Spherical	0.681	9.933	6.86
Spindle	0.725	10.150	7.14
Dumbbell	0.766	10.738	7.13

## Discussion

### Strength formation mechanism of cement-stabilized macadam with plastic aggregate

From a macroscopic point of view, doped waste plastic cement-stabilized macadam base is composed of pore structural units and solid structural units such as coarse aggregate, plastic coarse aggregate, fine aggregate. Although the strength of plastic coarse aggregate is not as strong as coarse stone, but plastic has better ductility and stronger deformation capacity. When the cement-stabilized macadam base is subjected to external forces, the plastic coarse aggregate in the mix deforms under the action of external forces, thus improving the ability of traditional base materials to resist deformation when subjected to loads, and to some extent improving the toughness of the semi-rigid base.

At the fine view level, the cement particles in the area away from the aggregate are more than that in the area around the aggregate during the molding process, forming the interface transition area with lower bond strength at the boundary of the aggregate. The strength of the interface transition area is much lower than the strength of the aggregate itself, and is also the weakest place in the internal strength of the mixture. The damage of the cement-stabilized macadam base mixed waste plastic can be seen as the expansion process of cracks. When the subgrade material is loaded, micro-cracks are formed and gradually expand as the load increases and eventually run through the entire road structure. The deformation of ordinary cement-stabilized macadam material is small when it is subjected to load, and the crack tip in the interface transition zone almost does not produce a plastic zone, causing a sudden and brittle damage. However, the crack tip in cement-stabilized macadam with plastic aggregate will produce a certain plastic zone by the plastic aggregate, which increases the load threshold for brittle damage, so that the toughness of the mixture is improved.

### Influence mechanism of geometric characteristics

#### Influence mechanism of roughness of surface texture

The influence of plastic coarse aggregate surface roughness on the bonding force is mainly in two aspects.

1. The surface texture and the trace depth of the plastic coarse aggregate are different. As shown in Fig. 7, when the two plastic coarse aggregate direct contact, the surface is uneven with each other, making the two nested in each other, increasing the internal friction. When the mixture is subjected to load, this interlocking interface has a higher resistance to failure. The rougher the surface of the plastic coarse aggregate, the more obvious the role of embedding, the better the performance of the mixture.

**Figure 7:**
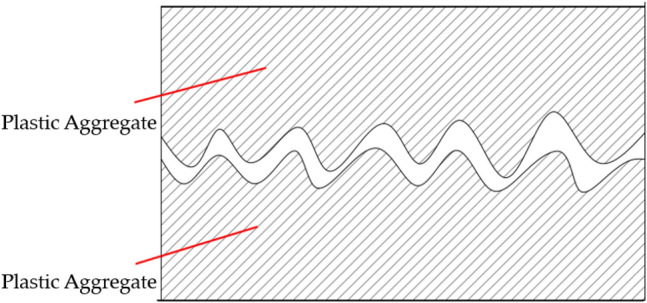
Embedding effect between the plastic coarse aggregate.
2. The rougher the surface of the plastic coarse aggregate, the larger the actual surface area, and the contact area of the gel is larger, enhancing the bonding strength of interface transition zone between the plastic coarse aggregate surface and gel. And when the surface of plastic coarse aggregate is smooth, the interface bonding strength is not high because of the hydrophobic nature of plastic.

In general, improve the surface roughness of the plastic coarse aggregate on the one hand can effectively increase the contact area between the plastic coarse aggregate and the gel, improve the bonding strength of the interface transition zone inside the mix; on the other hand, it can also enhance the embedding effect between the plastic coarse aggregate, increase the internal friction of the mix, and improve the anti-cracking performance of the mix.

#### Influence mechanism of shape of plastic aggregate

Among the cement-stabilized macadam base materials mixed with plastic aggregate, the dumbbell-shaped plastic coarse aggregate forms a higher-strength embedded structure with other plastic coarse aggregate or coarse stone due to its special aggregate shape. Its surface is also easier to adhere to the cement slurry, improving the bonding force of the weakest interface transition zone. And the special shape of the spindle-shaped plastic coarse aggregate also makes it easier to combine with the cement slurry, forming a dense skeleton between the plastic coarse aggregate and the coarse stone. After the skeleton voids are filled with fine aggregates, a high-strength mixture structure is formed, which improves the toughness and flexibility of the cement-stabilized macadam and inhibits the generation of cracks.

The spherical plastic coarse aggregate has the highest sphericity and the largest number of edges and corners. When the cement-stabilized macadam is mixed with plastic aggregate, the spherical plastic coarse aggregate has the best fluidity, which also leads to its more uniform dispersion in the mixture and will not form a weak area of concentrated strength of plastic coarse aggregate, which is conducive to the improvement of the overall strength of the mixture. After the mixture is formed, due to the large angularity of the spherical plastic coarse aggregate, a large internal friction resistance can also be generated between the aggregates, effectively preventing the movement of the particles, thereby forming a more stable structure. The prism of cylindrical plastic coarse aggregate is not obvious, and the surface texture is not rich, which leads to the unobvious inlay effect between aggregates.

## Conclusions

In this paper, plastic was used as an aggregate substitute to study the influence of geometry of plastic aggregate on the strength properties of cement-stabilized macadam. The main conclusions drawn from the study are as follows.

1. The surface of plastic aggregate was treated with Ra12.5–Ra25 to prepare cement-stabilized macadam specimens. Compared with the natural stone, the average splitting strength and average compressive strength of specimens incorporated with plastic aggregates were reduced. When the plastic coarse aggregate in the cement-stabilized macadam was 4%, 8%, 12%, 16%, 20%, and 24%, respectively, compared with aggregate with smooth surface texture, the compressive strength of the specimen with rough surface plastic coarse aggregate increased by 2.2%, 3.2%, 5%, 2.4%, 1.1%, and 0.5%, respectively. The splitting strength was increased by 3.9%, 7.3%, 10%, 9.7%, 4.8%, 5.7%, and the tension–compression ratio was increased by 1.6%, 4%, 4.8%, 7%, 3.4%, 5%.
2. The compressive strengths of specimens mixed with plastic aggregates ground into different geometries increase gradually from cylindrical, spherical, spindle-shaped to dumbbell-shaped. Compared with other types, the compressive strength of dumbbell-shaped plastic coarse aggregate cement-stabilized macadam is increased by 13.17%, 8.16%, and 5.81%, respectively, and the splitting strength of dumbbell-shaped plastic coarse aggregate cement-stabilized macadam is increased by 15.53%, 12.48%, and 5.66%, respectively. Plastic aggregate geometry significantly influences the compressive and splitting strengths of cement-stabilized macadam.
3. After adding the different plastic coarse aggregates, the tensile-compression ratio of the specimens mixed with spindle-shaped plastic coarse aggregates is the largest, which is slightly higher than that of the specimens mixed with dumbbell-shaped plastic coarse aggregates. Compared with the cement-stabilized macadam without plastic aggregate, the tensile-compression ratio is increased by 15.2%. The shape of the plastic coarse aggregate has a certain degree of influence on the tensile-compression ratio of the cement-stabilized macadam mixed with plastics.

In a conclusion, the results in this thesis show that it is feasible to make waste plastic into aggregates and apply them to the production of cement-stabilized macadam. The test results showed that the substitution rate, surface roughness and geometry of the plastic aggregate affected the performance of the cement-stabilized macadam. The incorporation of less than 20% plastic aggregate reduced the strength of the cement-stabilized macadam slightly, but improved their toughness significantly. The plastic aggregate with rough surface and dumbbell shape has the best improvement on the toughness of cement-stabilized macadam. This study provides a new approach to improve toughness of cement-stabilized macadam and a new reference for the engineering reuse of waste plastics.

## Data Availability

All data generated or analyzed during this study are included in this published article.
